# Prevalence of depression and associated factors among older adults at ambo town, Oromia region, Ethiopia

**DOI:** 10.1186/s12888-018-1911-8

**Published:** 2018-10-18

**Authors:** Yohannes Mirkena, Mebratu Mitiku Reta, Kibrom Haile, Zebiba Nassir, Malede Mequanent Sisay

**Affiliations:** 1 University of Gondar Teaching and Referral Hospital, Gondar, Ethiopia; 2 Department of Internal Medicine, School of Medicine, College of Medicine and Health Sciences, University of Gondar, Gondar, Ethiopia; 3 Amanuel Specialized Mental Health Hospital, Addis Ababa, Ethiopia; 4 Department of Epidemiology and Biostatistics, Institute of Public Health College of Medicine and Health Sciences, University of Gondar, Gondar, Ethiopia

**Keywords:** Prevalence, Depression, Older adults, Ethiopia

## Abstract

**Background:**

Depression is an important public health concern due to its devastating morbidity and mortality among older adults. The aim of this study was to assess the prevalence of depression and associated factors among older adults (age ≥ 60 years) in Ambo Town, Ethiopia, 2016.

**Methods:**

A community-based cross-sectional study was conducted among older adults in Ambo town from May to June 2016. Geriatric depression scale item 15 (GDS 15) was used to conduct face-to-face interviews with 800 study participants. Data were entered into Epi Info version 7 and analyzed using SPSS version 20. Descriptive statistics and multivariable logistic regression analysis were employed. Adjusted odds ratio (AOR) with a 95% confidence interval was used to calculate significance.

**Results:**

The prevalence of depression was found to be 41.8% [CI = 38.5%, 45.5%]. The multivariable logistic regression model revealed that female sex (AOR = 1.72; 95% CI = 1.12, 2.66), trading (AOR = 2.44; 95% CI =1.32, 4.57), living with children (AOR = 3.19, 95% CI =1.14, 8.93) and retirement (AOR = 3.94, 95% CI = 2.11, 7.35) were associated with depression among older adults.

**Conclusion:**

The prevalence of depression among older adult was found to be high. Due emphasis needs to be given to screening and treating depression, especially among older females, retired individuals, adults living with children and merchants.

## Background

Depression is a common mental health disorder in late-life and an important public health problem because of its devastating consequences at any given time in a community. Its recent global prevalence was 4.4% [1, 2]. Among all mentally ill individuals, 40% were diagnosed to have a depressive disorder [3]. Depression was the second global disease burden in 2010 and it is projected to be the first cause of years lived with disability (YLD) in 2020 [4]. It has been increasing in terms of both disability and mortality rates around the world. People with the depressive disorder have a 40% greater chance of premature death than their counterparts. The World Health Organization (WHO) estimated that the global depressive disorder among older adults ranged between 10 and 20%, depending on cultural situations; it affected nearly 300 million people in 2015 [5–8].

A community-based study done on an elderly population reported that depression was the most common mental health disorder in later life [9]. Although old age is not necessarily a time of sadness and depression, some elderly people face challenges that can be difficult to cope with effectively and experience distress, anxiety, demoralization, and loneliness. Most of the time, the clinical picture of depression in old age is masked by memory difficulties with distress and anxiety symptoms; however, these problems are actually secondary to depression [10–12]. Moreover, more older adults commit suicide, often through such indirect means as self-induced starvation or dehydration or failure to take medications prescribed for different reasons in the community [13, 14]. A population-based study showed that depression amplifies the functional disabilities caused by physical illness, interferes with treatment and rehabilitation and further contributes to a decline in physical and cognitive functioning of a person [15, 16]. It also has an economic impact on older adults due to its significant contribution to the rise of direct annual livelihood cost [17].

Several community-based studies showed that older adults experienced depression related complications, especially in low-income countries like Ethiopia [1, 18–21]. In addition, compared with other health services, evidence of depressive disorders tends to be relatively poor. Thus, the level of its burden among older adults is not well addressed in Ethiopia. Lack of adequate evidence about depression in older adults may be a factor that contributes to poor or inconsistent mental health care at community level [8, 22]. Therefore, the aim of this study was to investigate the prevalence of depression and associated factors among older adults in Ambo town, Oromia Region, Ethiopia.

## Methods

### Study design and setting

A community-based cross-sectional study was conducted in Ambo town, Oromia region, Ethiopia. The estimated number of households and the total number of the population in the town were 16,471 and 79,059 respectively, of which 39,553 (50.03%) were males. The town had one government hospital with a psychiatry clinic, which serves as an outpatient department. In addition, the town has 2 health centers, 3 health posts, 13 mediums, and 6 junior private clinics. However, none of these provides mental health care to the community. Older adults aged 60 years and above who were present at a time of data collection participated in the study. The United Nations uses 60 years to define old age and recommends the age range of 50 to 65 years to be used as cut off point by countries; additionally, the settings and contexts of countries play roles in determining old age [23]. In Ethiopia, old age starts at 60 the retirement age [24]. Householders from the selected zones who lived in the town for at least 6 months were eligible to participate. Those who were unable to communicate due to severe illness (individuals with hearing loss and aphasia, or difficulty to respond due to illness) were excluded from the study.

### Sample size and sampling procedure

The sample size was determined by using the single population proportion formula with the assumption prevalence (P) of depression, 47.5% of a study in Sudan [25], a 95% confidence interval and a 5% margin of error. The minimum sample size (n) required for the study was calculated by using a design effect of 2 and a 10% non-response rate. With this, the total sample size was found to be 844. The multi-stage sampling technique was used to select participants with the assumption of a homogenous population. The town had three sub-cities, each composed of different zones. Two sub-cities were selected by the lottery method and clustered into 18 zones. Forty percent of the zones and their households were also selected by the lottery method and every household in the selected zones was visited to interview participants.

### Data collection tools and procedures

Socio-demographic characteristics, substance use, clinical and psychosocial characteristics of older adults were collected through interviews. Substance use was assessed if the participant used substances like alcohol, khat, cigarettes and/or hashish in the preceding last 3 months. We also assessed clinical conditions that might contribute to depressions such as hypertension, diabetes and heart diseases. Geriatric Depression Scale item 15 (GDS-15) was used to assess the presence of depression among older adults. GDS item 15 has been extensively tested and validated in low and middle-income countries such as India, Nepal and other Asian countries [26–28]. This geriatric depression scale has been suggested by the Royal College of Physicians, the British Geriatric Society and the Royal College of general practitioners; the scale was recommended for screening depression in older people [29]. Depression was considered using a cutoff point greater than or equal to five. The Oslo-3 Social Support Scale (OSS-3) was used to collect social support characteristics. By adding the three items, those with 3–8 values were considered to have poor social support, while those with 9–11 and 12–14 values were considered to have intermediate and strong social support respectively. For this study, two different language experts translated the questionnaire from English into Afan Oromo and back to English to ensure consistency. A one day training was given to data collectors and the supervisor on basic data collection and interviewing techniques.

### Data processing and analysis

The completed questionnaire was manually checked for completeness. Then, it was coded and entered into Epi-Info version 7 and exported to SPSS version 20 for further analysis. Descriptive and summary statistics were used to explain the population with respect to the relevant variables. Variables with less than 0.2 *p*-value in the bivariate analysis were fitted to the multivariable logistic regression. Odds ratio with 95% CI were calculated and statistical significance was considered at *P*-values < 0.05 in the multivariable logistic regression.

## Results

### Socio-demographic characteristics of participants

A total of 800 participants were involved with a response rate of 94.8%. More than half (55%) were male. The mean age of the participants was 66.69 (SD ± 6. 18) years. Nearly half, 359 (44.9%) were married and living with their spouses, while 174 (21.8%) were living only with their children. More than half, 437 (54.6%) of the participants had a secondary or higher level of education. Among the respondents, 569 (71.1%) were Orthodox Christians. One-third of the respondents were daily laborers. Of the respondents, 103 (12.9%) and 102 (12.8%) were merchants and retired people, respectively (Table 1).

**Table 1:** Socio-demographic characteristics of older adults at Ambo Town, Oromia region, Ethiopia, 2016 (*n* = 800)

Variables	Categories	Prevalence	Depression		Frequency	Percent
			Yes	No		
Sex	Female	0.46	167	193	360	45.0
	Male	0.38	167	273	440	55.0
Age	60–64	0.28	*103*	*271*	374	46.8
	65–69	0.44	*95*	*119*	214	26.8
	70–74	0.59	*68*	*47*	115	14.4
	75+	0.70	*68*	*29*	97	12.0
Ethnicity	Oromo	0.39	*266*	*413*	679	84.9
	Amhara	0.55	*57*	*46*	103	12.9
	Others^a^	0.61	*11*	*7*	18	2.2
Educational Status	Secondary & above	0.37	162	275	437	54.6
	Primary	0.55	128	105	233	29.1
	Not Educated	0.42	44	86	130	16.3
Marital Status	Married	0.36	128	231	359	44.9
	Widowed	0.39	129	201	330	41.2
	Single	0.64	7	4	11	1.4
	Separated/ divorced	0.70	70	30	100	12.5
Religion	Orthodox	0.34	*196*	*373*	569	71.1
	Protestant	0.59	*99*	*68*	167	20.9
	Others^b^	0.61	*39*	*25*	64	8.0
Occupation	Employed (Government & private organizations)	0.32	38	79	117	14.5
	Retired	0.71	72	30	102	12.8
	merchant (self-employed)	0.59	61	42	103	12.9
	Housewife	0.43	91	123	214	26.8
	Jobless and Daily laborer	0.27	72	192	264	33.0
Income (EBR)	<735	0.41	*70*	*101*	171	21.4
	735–1176	0.43	*209*	*281*	490	61.2
	>1176	0.40	*55*	*84*	139	17.4
Living arrangement	Spouse	0.35	125	230	355	44.4
	Children	0.75	130	44	174	21.8
	Alone	0.37	56	94	150	18.8
	Others^c^	0.19	23	98	121	15.1

^a^Tigre, Gurage ^b^Muslim, Catholic, Wakefata, ^c^relatives

The majority, 743 (92.9%) of the respondents had no known self or family history of mental illness. Four hundred forty-nine (56.1%) had a history of other medical illnesses, of which 171 (38%) were diabetic. Less than half of the participants, 356 (44.5%) had strong social support, while 223 (27.9%) had poor help (Table 2). The prevalence of depression among older adults was found to be 41.8% [95% CI (38.5, 45.5)].

**Table 2:** Clinical, social support and substance use of the study participants at Ambo Town, Oromia region, Ethiopia, 2016 (*n* = 800)

Variables		Frequency (n)	Percentages (%)
Mental illness	Yes	57	7.1
	No	743	92.9
Family history of mental illness	Yes	61	7.6
	No	739	92.4
Medical illness	Yes	449	56.1
	No	351	43.9
Social support	poor	223	27.9
	Intermediate	221	27.6
	Strong	356	44.5
Substance use (current)	Yes	134	16.8
	No	666	83.2

### Factors associated with depression

This study showed that retirement, living with children, trading and female sex were significantly and independently associated with depression. However, depression had no significant association with income, substance use, clinical and psychosocial characteristics. Elderly female were 1.7 times more likely to have depression compared to males [AOR = 1.72, 95% CI (1.12, 2.66)]. Retired older individuals were almost four times more likely to have depression than their counterparts [AOR = 3.94, 95% CI (2.11, 7.35)]. Older merchants were 2.4 times more likely to be depressed than employees [AOR = 2.44, 95% CI (1.32, 4.57)]. Older individuals living with children were 3.2 times more likely to have depression than those living with their spouses [AOR = 3.19, 95% CI (1.14, 8.93)] (Table 3).

**Table 3:** Multiple Logistic regression analysis of depressive disorder among older adults at Ambo Town, Ethiopia, 2016 (*n* = 800)

Variables	Categories	Depression (*n* = 800)		COR (95% CI)	AOR (95% CI)	*P* value
		Yes	No			
Sex	Female	167	193	1.41 (1.07,1.88)	1.72 (1.12,2.66)	0.013
	Male	167	273	1	1	
Marital status	Married	128	231	1	1	
	Widowed	129	201	1.16 (0.85,1.58)	1.28 (0.45,3.63)	0.52
	Single	77	34	3.16 (0.91,10.00)	4.78 (0.95,23.99)	0.049
	Separated/divorced	70	30	4.21 (2.61,6.80)	2.75 (0.92,8.24)	0.29
Education	Secondary and above	162	275	1	1	
	Primary	128	105	2.07 (1.50,2.86)	1.24 (0.80,1.92)	0.15
	Not educated	44	86	0.87 (0.58,1.31)	0.69 (0.39,1.21)	0.209
Occupation	Employed (Government & Private Organization)	38	79	1	1	
	Retired	72	30	4.99 (2.81,8.87)	3.94 (2.11,7.35)	<0.001
	Merchant (Self-employed)	61	42	3.02 (1.74,5.24)	2.44 (1.32,4.57)	0.009
	Housewife	91	123	1.54 (0.96,2.47)	0.86 (0.44,1.67)	0.712
	Others^a^	72	192	0.78 (0.49,1.25)	0.92 (0.50,1.69)	0.701
Living arrangement	Spouse	125	230	1	1	
	Children	130	44	5.44 (3.63,8.15)	3.19 (1.14,8.93)	0.028
	Others^b^	79	192	0.76 (0.54,1.06)	0.57 (0.20,1.64)	0.281

^a^jobless, daily laborer, ^b^alone, relatives

## Discussion

In this study, the prevalence of depression among older adults was 41.8% [95% CI: 38.5%, 45.5%]. The finding was in line with that of a community based cross-sectional study done in Sudan (41.1%) [25], Saudi Arabia (39%) [30] and India (42.7%) [31]. However, this finding was higher than the finding of a systematic review in a Caucasian population, where 1 to 16% of the older adults had depression according to both community and institution based study [32]. This variation may be due to the difference in study areas and socioeconomic status of participants. Conversely, our finding is in line with the finding of a systematic review done on a hospitalized Caucasian population, where 14–42% had depression. The finding of this study is also higher than the finding of a community-based survey in Thailand, where a 12-item questionnaire of Thai validated Euro-Depressions scale was used and a 28.5% depression prevalence was noted in old age [33]. The difference could be due to variations in the tools used to screen depression. This result is also higher than that of a meta-analysis conducted in nine European countries, where a pooled prevalence of depression was 12.3% in older adults [34]. This disparity may be due to social-cultural and economic difference. Again, the finding is also higher than that of a community-based survey conducted in Singapore (13.4%) [35]. The variation may be due to differences in tools, since the study in Singapore used the Geriatric Mental State (GMS) to screen depression. Moreover, compared to an institution based study in Saudi (17%), our finding is higher [36]. This discrepancy may be due to the difference in the tool they used, the study setup and socio-cultural variations among the study participants. This finding is also higher than that of a community-based study in India (12.7%) and Mexico (23.7%) [37, 38]. Again, this variation may be due to differences in tools used to measure depression. Geriatric depression (ICD-10) and the seven-item version of the Center for Epidemiologic Studies Depression Scale (CES-D) were used in India and Mexico, respectively, beside the socio-cultural and economic differences. The finding of our study was also higher than the finding in the Netherlands (14.9%) [39]. The possible reason may be the difference in the tool they used and socioeconomic variations. In addition, ages 55–85 were included in the Netherland study, whereas age 60 and above were used as a cutoff point in our study. Our finding was higher than the finding of a community-based study conducted among South African older adults 4% [40]. This variation may be due to differences in age of participants in addition to the socio-cultural variations. In the South African study, ages 50 and older were included. The finding of our study was lower than those of other institutions based cross-sectional studies done in Brazil, India, Portugal, and elderly Japanese which reported 49.76%, 53.75%, 61.40% and 57.2%) respectively [41–43]. This variation may be due to the differences in the study setups. The elderly people living in institutions could be more depressing due to changes in the environment after living in private dwelling for a long time. Like our study, research in low and middle income countries (Sudan, Saudi Arabia, India) showed a higher prevalence of depression among the elderly than among the upper-middle and high income countries such as South Africa, Thailand, Singapore, Mexico, and the Netherlands [25, 30, 33, 35, 38–40]. This variation may be due to the impact of socioeconomic status; additionally, people in developed countries may have more access to mental health care and support before they develop problems. Previous studies also revealed that the associations between socio-demographic characteristics and depression among older adults were based on gender, marital status, and occupation [25, 44, 45]. The finding of this study also indicates supports that the same socio-demographic characteristics are significantly and independently associated with depression. Elderly female adults were 1.7 times more likely to have depression than males (AOR = 1.72, 95% CI: 1.12, 2.66). This was in line with the finding in Sudan, Saudi Arabia and India, where females were more affected by depression than males [25, 30, 36, 42, 46]. In addition, this finding was also supported by a systematic review and meta-analysis conducted in 2003 [47]. This finding was also supported by that of another cross-sectional study conducted in Italy [48]. This may be because women bear the burden of household responsibilities in addition to their economic dependency on men, especially in low-income countries.

Retirement was significantly associated with depression in older adults. Retired older adults were 4 times more likely to have depression compared to elderly working individuals [AOR = 3.94, 95% CI (2.11, 7.35)]. This is in line with the finding of a household survey in Sudan, Khartoum [49]. The result was also supported by the finding of a Korean Longitudinal Study of Aging [50]. This may be because retired individuals may not have adequate opportunity to interact with other people to share ideas and feelings. Elderly people may feel like they are isolated and have no support. Furthermore, such feelings might contribute to the development of depression.

Merchant older adults were 2.4 times more likely to develop depression than government employees [AOR = 2.44, 95% CI (1.32, 4.57)]. This was in line with a finding in Malaysia [51]. This may be due to the nature of the work, which involves economic uncertainty. Additionally, depression may visit the elderly when they try to manage their businesses based on their own and work to sustain their business even after they no more able to do so. This study also found that older adults who were living with their children were 3.2 times more likely to develop depression compared to those living with spouse [AOR = 3.19, 95% CI (1.14, 8.93)]. This is supported by another study conducted in India [42] showing that feelings of dependency may lead to the development depression. In previous studies, self and family history of mental illness, the absence of formal education and low income were significantly associated with older adult depression [44, 52, 53]. In this study, however, these variables had no significant association with such depression. Other studies also showed that being single, separation and divorce were significantly associated with older adult depression, but in our study, these variables had no statistically significant association [25, 54]. The disparity could be due to differences in sampling techniques, sample size, measurement tools and other socio-cultural variations among participants.

## Conclusion

The finding of this study showed that the prevalence of depression among older adults was high. Retirements, merchant in occupation, living with children and female sex were significantly associated with depression. Greater emphasis has to be given in identifying and treating depression, especially the retired adults, merchants, adults living with children and females. Further studies with solid study design and other important variables need to be considered.

## Abbreviations

ADLs: Activities of Daily Living
AOR: Adjusted Odds Ratio
COR: Crude Odds Ratio
CTIDHM: Clinical Tropical Infectious Disease and HIV Medicine
DALYs: Disability Adjusted Life Years
GDS: Geriatric Depression Scale
ICCMH: Integrated Clinical and Community Mental Health
LAMIC: Low and Middle-Income Countries
MDD: Major Depressive Disorder
SPSS: Statistical Package for Social Science
UAE: United Arab Emirates
UK: United Kingdome
UN: United Nation
WHO: World Health Organization
YLD: Years Lived with Disability
